# Supplementary material for: Idiopathic Bell′s Palsy in a Patient With Metastatic Lung Adenocarcinoma Receiving Nab‐Paclitaxel and Pregabalin: A Rare Clinical Observation
Source: Case Rep Oncol Med. 2026 May 4;2026:4818820. doi: 10.1155/crom/4818820 (PMC13136843; doi:10.1155/crom/4818820)
Supplement: Supplementary file 1 — Supporting Information Additional supporting information can be found online in the Supporting Information section. [file CROM-2026-4818820-s001.docx]

**Title:** I**diopathic Bell's palsy in a patient with metastatic lung adenocarcinoma receiving nab-paclitaxel and pregabalin: a rare** clinical observation

**Authors:** Vaishnavi Bamane^1^, Shrutika Thakre^1^, Aditya Dhanawat^1^, Ganesh Chepuri^1^, Vikram Gota^1,3^, Amit Joshi^2,3^, Manjunath Nookala Krishnamurthy^1,3^

**Corresponding author:** Manjunath Nookala Krishnamurthy

**1: Department of Clinical Pharmacology, Advanced Centre for Treatment, research and Education in Cancer, Tata Memorial Centre, Kharghar, Navi Mumbai**

**2: Department of Medical Oncology, Advanced Centre for Treatment, research and Education in Cancer, Tata Memorial Centre, Kharghar, Navi Mumbai**

**3: Homi Bhabha National Institute, Anushaktinagar, Bombay**

**Abstract:**

**Background:** Chemotherapy drugs are known to cause peripheral neuropathy of varying severity. However, Bell's palsy, which is an acute lower motor neuron facial nerve palsy, is rarely observed in oncology patients receiving chemotherapy. **Case presentation:** We report a case of Bell's palsy in a 74-year-old male metastatic lung adenocarcinoma patient while on treatment with Nab-paclitaxel and pregabalin. Temporal association, diagnostic workup, and management are discussed in this case report to highlight the importance of differentiating neurological adverse effects in cancer therapy. **Conclusion**: The most likely causative factor remained nab-paclitaxel-induced Bell's palsy in our patient. However, viral reactivation due to immunosuppression resulting from drug toxicity cannot be completely ruled out.

**Keywords:** nab-paclitaxel, Bell's palsy, pregabalin, Herpes Simplex Virus

Manuscript preprint is available in Authorea, which is an open repository platform and bio archival (Wiley) for open view and review by others, allowing public access and feedback. This manuscript is unpublished and has not undergone peer-reviewed journal publication.

**Background:**

Bell's palsy is the most common cause of acute unilateral peripheral facial nerve paralysis, characterized by the sudden onset of facial muscle weakness or paralysis. Although the exact aetiology remains unclear, viral infections – particularly herpes simplex virus type 1 (HSV-1)—are implicated in many cases, along with immune-mediated mechanisms and ischemic events affecting the facial nerve (cranial nerve VII) (1). While Bell's palsy is relatively prevalent in the general population, with an annual incidence of approximately 20–30 cases per 100,000 persons, its occurrence in patients undergoing systemic chemotherapy is rare and infrequently reported in the literature (2, 3).

In cancer patients, neurological complications such as chemotherapy-induced peripheral neuropathy (CIPN) are well-documented, particularly with agents such as taxanes (paclitaxel, docetaxel), platinum analogs, vinca alkaloids, proteosome inhibitors, etc (4). However, cranial neuropathies, and specifically idiopathic facial nerve palsy, have not been widely associated with taxane-based regimens. Pregabalin, a commonly prescribed agent for neuropathic pain, modulates presynaptic voltage-gated calcium channels in the central nervous system but has not been conclusively linked to facial nerve dysfunction.

We observed a case of idiopathic Bell's palsy in a 74-year-old metastatic lung adenocarcinoma patient receiving nab-paclitaxel and pregabalin. Pregabalin was prescribed for peripheral neuropathy management. The temporal correlation between drug administration and symptom onset and radiologic exclusion of metastatic or vascular causes emphasizes the need for increased awareness of acute neurological presentations in oncology patients.

**Case presentation:**

On August 29, 2024, a 74-year-old man presented to a tertiary care cancer hospital outpatient department with metastatic adenocarcinoma of the left lung. He was a known case of hypertension, for which he was on treatment with the tablet amlodipine 2.5 mg daily. Patient's past medical history revealed that he is on tablets, ecosprin (75 mg/day), and rivaroxaban (20mg) for right internal jugular vein (IJV) thrombosis, which was diagnosed during the cancer diagnostic workup. The patient was also on Folic Acid (Folvite 5 mg) tablets for anemia treatment. Notably, he had no addictions, no family history of cancer, and recovered from COVID-19 in 2021 without complications.

For the cancer management, nab-paclitaxel (380 mg IV, every 3 weeks) and Carboplatin (330 mg IV, every 3 weeks) were initiated on September 07, 2024. Initially, the regimen continued for eight cycles; nab-paclitaxel continued for three more cycles until April 15, 2025. During this treatment period, as early as the third cycle, the patient developed early features suggestive of peripheral neuropathy (CIPN). By March 2025 (ninth cycle), CIPN progressed to Grade 1, and Grade 3 by the 11^th^ cycle, necessitating the addition of Pregabalin 75 mg at bedtime, in an attempt to alleviate burning neuropathic discomfort in his lower limbs. Subsequently, the patient observed that the left eye could not be closed entirely, and there was drooping of the angle of the mouth to the right side. Clinical evaluation on April 21, 2025, revealed that the patient developed left facial nerve LMN palsy. CT brain performed on April 22 showed no signs of metastasis, bleeding, or infarction in the brain. Causality assessment by Naranjo and SNG assessment scales (with the existing information) revealed that the adverse event was probably caused by nab-paclitaxel and the subsequent cycles of nab-paclitaxel were withheld. No other non-invasive or non-radiation–based diagnostic modalities (high resolution ultrasonogrphy, electrophysiological tests) were further considered. On subsequent follow-up in medical oncology OPD on May 06, 2025, LMN symptoms of the facial nerve were stable but were not completely resolved, and he still had facial weakness. An MRI of the brain done on May 07, 2025, ruled out any central causes of facial nerve palsy, such as brain metastasis or infarction.

As there was no radiological evidence to suggest a structural cause, nor any other systemic triggers, the clinical diagnosis of idiopathic Bell's palsy was made and according to House–Brackmann grading system assessment the severity Grade was IV (moderately severe dysfunction). However, the patient was started on tablet acyclovir 400 mg thrice daily for one week from May 08, 2025, along with physiotherapy to stimulate facial muscles and prevent synkinesis.

Over the next few weeks, a gradual improvement in his clinical features was noted. The right-sided facial droop slowly began to resolve, and he regained better voluntary control of facial expressions. Follow-up evaluation on July 02, 2025, revealed that although his facial weakness was reduced, left eye synkinesis was present with peripheral neuropathy symptoms of the upper and lower limbs and now the House–Brackmann grading system assessment revealed Grade 3 severity (moderate dysfunction).

Given this, the patient was advised to take the tablet duloxetine for neuropathy, and chemotherapy was still kept on hold. The causality assessment score for the drug nab paclitaxel was 5 (possible)

**Clinical Timeline – Flow Chart**

Patient: 74-year-old male; Diagnosis: Metastatic adenocarcinoma of the lung (primary site)

**Table 1: Cycle-wise event/ intervention flow chart**

| **Date** | **Event / Intervention** |
| --- | --- |
| Commencement of 1^st^ cycle of nab-paclitaxel + carboplatin 07/09/2024 | Nab-paclitaxel + Carboplatin: Three weekly treatment regimens were planned, and 1^st^ cycle was administered. |
| After completing nine cycles, by February 02, 2025 | - Developed Grade 1 Peripheral Neuropathy (PN) - Grade 3 Anemia - Carboplatin discontinued |
| After 11 cycles are completed by April 15, 2025 | - Developed Grade 3 PN - New-onset Left LMN Facial Palsy |
| Workup | - MRI Brain with contrast: No metastasis - All other lab parameters are within normal limits - Started on Acyclovir 400 mg TID from 8/5/25 and Tab duloxetine 20 mg HS from 2/7/25 |
| Conclusion | - Diagnosed as Idiopathic Bell's palsy. According to House–Brackmann grading system assessment- Grade was IV (moderately severe dysfunction).  - Continued Acyclovir till resolution  - Tab. Duloxetine was started for neuropathy |

**Differential diagnosis for Bell's palsy in this patient:** Based on the case presentation of acute unilateral lower motor neuron facial nerve palsy while on n**ab-paclitaxel** in a 74-year-old male with metastatic lung adenocarcinoma**.** The differential diagnosis for Bell's palsy is detailed in Table 2.

**Discussion:** Bell's palsy, an acute idiopathic peripheral facial nerve paralysis, is a relatively common neurological condition in the general population; its occurrence in oncology patients undergoing chemotherapy remains rare and poorly documented. This case presents an unusual occurrence of idiopathic Bell's palsy in a 74-year-old male with metastatic lung adenocarcinoma receiving nab-paclitaxel and pregabalin for peripheral neuropathy.

**Table 2: Differential diagnosis for Bell's palsy with its mechanism and justification.**

| **Differential diagnosis (DD)** | **Mechanism** | **Is the DD "for" or "against" our case** | **Justification** |
| --- | --- | --- | --- |
| **Herpes Zoster Oticus (Ramsay Hunt Syndrome)** | Reactivation of varicella-zoster virus in the geniculate ganglion | Against | Usually presents with LMN facial nerve palsy, plus vesicular rash around the ear or in the oral cavity. Our patient did not have any vesicular rash along the facial nerve track. |
| **Metastatic Involvement of the Facial Nerve** | Direct compression or infiltration of the facial nerve by tumor | Against | CT and MRI brain imaging show no evidence of brain metastasis, which can cause direct compression of the facial nerve. |
| **Otitis Media / Mastoiditis** | It can lead to facial nerve compression. | Against | No otologic symptoms were present for our patient. |
| **Ischemic Stroke** | Pontine Infarct | Against | Usually causes central facial weakness, and typically spares the forehead.  MRI shows no infarct, and the pattern of facial weakness is consistent with a lower motor neuron lesion (forehead involved) |
| **Chemotherapy-Induced Cranial Neuropathy** | Drug toxicity on the nerves | “For”  and  “Against” | 1. Rarely, high doses of taxane can cause neuropathy (5).   The dose of nab-paclitaxel that was prescribed for our patient was within the prescribed dose range.   1. Nab-paclitaxel neurotoxicity is usually peripheral (6). |
| **Pregabalin-Induced Neurological Effects** |  | “For”  and  “Against” | Pregabalin pharmacovigilance data have shown that it can cause drug-induced hyponatremia, which can lead to cerebral edema and diverse neurological manifestations with an odds ratio (ROR = 2.5; 95% CI: 1.4–4.5) (7).  Pregabalin-induced neuropathy is preceded by various clinical symptoms, which are entirely lacking in our patient. |
| **Paraneoplastic Syndrome** | Hormones or chemokines produced by the ectopic sites | Against | - 1. Neurological paraneoplastic syndromes can rarely involve cranial nerves.   2. We did not evaluate any specific autoantibodies. No systemic signs and symptoms were noted in this case, but the absence of other neurologic deficits and imaging findings makes paraneoplastic syndromes less likely. |
| **Lyme Disease** |  | Against | Can present with facial palsy (often bilateral), though uncommon in India. Our patient has no travel exposure and has not reported tick exposure. Furthermore, the patient is not having any systemic symptoms. |
| **Guillain-Barré Syndrome (Miller Fisher Variant)** | Ascending paralysis | Against | May present with bilateral facial palsy and ataxia. However, the patient has no ascending weakness, areflexia, or cranial polyneuropathy reported here. |
| **Eagle’s syndrome** | Involves an elongated styloid process (>30 mm) or ossified stylohyoid ligament compressing or irritating adjacent cranial nerves (V, VII, IX, X), carotid arteries, or the jugular vein | Against | PET CT brain imaging (that was previously performed to rule out metastasis or infarction), did not demonstrate elongation of the styloid process.  No calcified stylohyoid ligament or anatomical abnormalities suggestive of Eagle’s syndrome were noted. |

Systemic and biochemical factors may contribute to the pathogenesis of bell’s palsy in which case bilateral presentation is less common than the unilateral presentation^8^. Facial nerve traverses through the narrow Fallopian canal, any minor inter individual anatomical variations in the path of the facial nerve (in the soft tissue structures/bony structures or mucosal edema) can lead to increased susceptibility to facial palsy in the background of the insult caused by systemic changes^9^. All of these subtle anatomical variations may not be detectable by the routine imaging techniques, which may explain why imaging did not reveal any structural abnormalities, if any, in this patient.

Microvascular insults do cause the bell’s palsy; however, the patient did not have any predisposing factors such as long-standing diabetes mellitus or other neuropathy features. However, considering the history of hypertension and internal jugular vein thrombosis in this patient, there is a chance of increased susceptibility of vascular injury.

Transient immune suppression during chemotherapy might have facilitated reactivation of latent neurotropic viruses such as Herpes Simplex Virus Type 1 within the geniculate ganglion, leading to localized neuritis, resulting in localized inflammation and edema of the facial nerve within the fallopian canal. Because viral reactivation typically occurs unilaterally, this mechanism could explain the unilateral facial nerve involvement observed in our patient. Literature evidence regarding the laterality distribution of the bell’s palsy shows that unilateral presentation is more common than bilateral presentation ^10,11^.

The decision not to pursue additional CT-based imaging in this patient was made in the context of ongoing oncologic management, where patients are frequently exposed to regular multiple radiologic investigations during the course of cancer diagnosis, treatment, and follow-up. Considering the cumulative radiation exposure, further CT imaging was deferred after clinical assessment and multidisciplinary discussions. In addition, there were no noted features suggestive of bony abnormalities in the previous CT scans that could cause by themselves cause the facial palsy.

Nevertheless, structural evaluation remains an important consideration when assessing facial nerve palsy in oncology patients to exclude compressive, metastatic, or treatment-related causes. In this context, emerging radiation-free imaging techniques may provide useful complementary diagnostic information that we missed due to non-availability at our institute. Recent studies have highlighted the potential role of high-resolution ultrasonography (HRUS) as a diagnostic and prognostic tool in Bell’s palsy. HRUS can help assess facial nerve swelling, inflammatory changes, and may assist in predicting recovery outcomes. HRUS based approaches for facial nerve evaluation could represent a useful adjunct or future direction, particularly in situations where radiation exposure is undesirable. In addition, recent literature has described ultrasound-guided integrated musculoskeletal and vascular landmark approaches for accessing and visualizing the facial nerve trunk^10,11^. Such techniques may offer a valuable adjunct in situations where additional CT-based imaging is undesirable due to concerns regarding cumulative radiation exposure.

Repetitive peripheral magnetic stimulation (rPMS) delivers pulsed magnetic fields that induce electric currents in peripheral nerves is being tried in the bell’s palsy as it enhances facial nerve excitability and conduction, improve microcirculation and reduce edema, promote neuroplasticity and muscle reactivation, and potentially reduce synkinesis during recovery. As we did not have rPMS in our institute, we managed the patient with the anti-viral drug and followed up regularly. However, we did not provide the adjunct treatment with rPMS. This case represents the importance of maintaining a broad differential when new neurological symptoms develop during cancer treatment. Recognizing Bell's palsy early, even in oncology settings, allows for timely intervention and better outcomes. Prompt administration of acyclovir and physiotherapy can contribute to gradual clinical improvement, aligning with standard management recommendations.

**Conclusion:** As most of the central and structural causes were effectively ruled out by imaging, excluding other causes of facial nerve palsy, and given the temporal association of drug administration, the most likely causative factor for Bell's palsy in our patient could be nab-paclitaxel, possibly precipitated by immunosuppression due to drug toxicity. A possibility of viral reactivation can also be considered as a causative factor for Bell’s palsy.

**Take‑away lesson:** There should be close follow-up of the patients who are on treatment with anti-cancer medications. Patients should be monitored for the occurrence of rare neurological adverse events such as Bell’s palsy. Diagnosis of Bell’s palsy should be made idiopathic after excluding other causes.

**Patient perspective:** When Bell’s palsy develops in a patient, the patient may worry that a brain stroke might have happened to them and may fear that there can be paralysis of one half of the body. However, prompt reporting to the consultant and proper evaluation by the consultant will reassure them that the condition is limited to half of the face and that the causative factor can be identified by radiology imaging and other lab investigations.

**List of abbreviations:**

**CIPN –** chemotherapy-induced peripheral neuropathy

**LMN –** lower motor neuron

**PN** – peripheral neuropathy

**IJV –** Internal Jugular Vein

**HSV** – herpes simplex virus

**Declarations:** This case report received formal IRB exemption from the Institutional Ethics Committee (Exemption Reference No: 139/2025).

**Consent for publication-** Written informed consent will be obtained from the patient to publish the case report and any accompanying images or clinical information. We told the patient that the case report does not contain his identifier, which would disclose his identity.

**Availability of data and materials-** The data that support the findings of this study are available from the corresponding author upon reasonable request

**Competing interests-** The authors declare that they have no competing interests.

**Funding**- No funding was received for this research.

**Authors' contributions-** VB, ST, GC, collected the clinical data; VB, MN drafted the manuscript. MN, AJ, AS, and VG contributed to the interpretation and critical revision of the manuscript. All authors read and approved the final manuscript.

**Acknowledgements-** We thank the patient for their cooperation and willingness to share their medical history for academic purposes. Manuscript preprint is available in Authorea, which is an open repository platform (Wiley) for open view and review by others.

**References:**

1. Peitersen E. Bell's palsy: The spontaneous course of 2,500 peripheral facial nerve palsies of different etiologies. Acta Otolaryngol. 2002. <https://doi.org/10.1080/000164802760370736>
2. Tiemstra JD, Khatkhate N. Bell's palsy: Diagnosis and management. Am Fam Physician. 2007;76(7):997-1002.
3. Holland NJ, Weiner GM. Recent developments in Bell's palsy. BMJ. 2004;329(7465):553-557. <https://doi.org/10.1136/bmj.329.7465.553>
4. Hershman DL, Lacchetti C, Dworkin RH, et al. Prevention and management of chemotherapy-induced peripheral neuropathy in survivors of adult cancers: American Society of Clinical Oncology Clinical Practice Guideline. J Clin Oncol. 2014;32(18):1941-1967. <https://doi.org/10.1200/JCO.2013.54.0914>
5. Lee RT, Oster MW. Bilateral facial nerve palsy secondary to the administration of high-dose paclitaxel. Ann Oncol. 1999;10(8):997-1001. <https://doi.org/10.1023/a:1008380800394>
6. Postma TJ, Vermorken JB. Paclitaxel-induced neuropathy. Ann Oncol. 1995;6(5):489-494. <https://doi.org/10.1093/oxfordjournals.annonc.a059220>
7. Estévez Asensio L, et al. Drug-induced hyponatraemia and possible related signals: Analysis of 659 cases reported to the Spanish Pharmacovigilance System and disproportionality analysis. Med Clin (Barc). 2024. <https://doi.org/10.1016/j.medcli.2024.07.021>
8. Dalrymple SN, Row JH, Gazewood J. Bell Palsy: Rapid Evidence Review. Am Fam Physician. 2023 Apr;107(4):415-420. Erratum in: Am Fam Physician. 2024 Feb;109(2):105. PMID: 37054419
9. Singh A, Deshmukh P. Bell's Palsy: A Review. Cureus. 2022 Oct 11;14(10):e30186. doi: 10.7759/cureus.30186. PMID: 36397921; PMCID: PMC9648613.
10. Lee RT, Oster MW, Balmaceda C, Hesdorffer CS, Vahdat LT, Papadopoulos KP. Bilateral facial nerve palsy secondary to the administration of high-dose paclitaxel. Ann Oncol. 1999 Oct;10(10):1245-7. doi: 10.1023/a:1008380800394. PMID: 10586344.
11. Minatani N, Kosaka Y, Sengoku N, Kikuchi M, Nishimiya H, Waraya M, Enomoto T, Tanino H, Watanabe M. [A case of facial nerve palsy induced by nab-paclitaxel]. Gan To Kagaku Ryoho. 2013 Nov;40(12):2375-7. Japanese. PMID: 24394117.
